# Structural, Magnetic, and Dielectric properties of Sr_4_Fe_6_O_13_ ferrite prepared of small crystallites

**DOI:** 10.1038/s41598-020-61460-x

**Published:** 2020-03-18

**Authors:** A. A. Azab, A. M. Mansour, G. M. Turky

**Affiliations:** 1 Solid State Electronics Laboratory, Solid State Physics Department, Physical Research Division, National Research Centre, 33 El-Bohouth St., Dokki, Giza, P.O. 12622 Egypt; 2 Microwave Physics & Dielectrics Department, Physical Research Division, National Research Centre, 33 El Bohouth St., Dokki, Giza, P.O. 12622 Egypt

**Keywords:** Ferroelectrics and multiferroics, Magnetic properties and materials

## Abstract

A stable Sr_4_Fe_6_O_13_ was prepared as small crystallites by auto-combustion of a sol-gel in air followed by annealing the later at pertinent temperatures. A green sample, as annealed at elevated temperatures, yields a single Sr_4_Fe_6_O_13_ phase of tailored magnetic properties. The structural, morphological, magnetic and electrical properties were investigated by X-ray diffraction, transmission electron microscopy, vibrating sample magnetometer, and broadband dielectric spectrometer. Hard magnetic Sr_4_Fe_6_O_13_ properties arise with saturation magnetization M_s_ = 12.4 emu/g, coercivity H_c_ = 3956.7 Oe and squareness 0.512. Studies made at low temperatures reveals M_s_ decreasing on increasing temperature from 17.5 emu/g at 85 K down to 12.4 emu/g at 305 K, while H_c_ rises from 1483 Oe at 85 K to 1944 Oe at 305 K. The ac-conductivity follows the Jonscher relation. The dc-conductivity at high temperatures/low frequencies exhibits a plateau and it depends linearly on a characteristic frequency according to the Barton-Nakajima-Namikawa) relation.

## Introduction

Perovskites are compounds of a structural formula ABC_3_, where A represents a rare earth, alkaline earth, alkali or large ions such as Pb^2+^, Bi^3+^, B represents a transition metal ion and C represents O, Fl, Cl, I etc^1^. A cation may be monovalent like Li, Na, K, divalent like Ca, Ba, Sr or trivalent like La, Nd, Pr, which is cubo-octahedrally coordinated to 12 O^2−^ ions, while B cation, such as Ti, Ni, Fe, Co, or Mn is octahedrally coordinated to 6 O^2−^ ions^2^. Recently, several investigations performed on a Sr-Fe-O structure reveal its amazing structural and physical features such as cheap price, high magnetic anisotropy, high Curie temperature, a significant magnetization of saturation and remarkable chemical and corrosion resistance^3^. The reason certainly presented by oxygen-lacking perovskites and by Ruddlesden-Popper (RP) type structure that possess a desirably negative magnetoresistance (-ve MR)^4–7^. A Sr-Fe-O system includes many types of perovskites and perovskite derivatives of widely varied crystalline and magnetic features^8–12^. These types of substances are constructed based on a K_2_NiF_4_ shape and involve slab segments of SrFeO_3_ and SrO, where SrFeO_3_ is resulting from a KNiF_3_ cubic perovskite of K_2_NiF_4_, and SrO is matching to a NaCl-class KF^8^. They characteristically contain paramagnetic Fe^4+^ ions.

In particular, a stoichiometric compound Sr_4_Fe_6_O_13_ has a construction of a perovskite or its derivatives^8^. It is observed that a Sr_4_Fe_6_O_13±δ_ construction is a highly stable single-phase compound and it exhibits significant conductivity of combined-ions and electrons types^13^. That is composed of altered sections through a Sr-Fe-O layer (b-axis) and dual slabs of FeO in FeO_5_ polygons^14–16^. As a result, it owes an anisotropic shape in it conducts through O^2−^ ions and vacancies across the a-c planes^17^. Actually, creation of O^2−^ empty sites and interstitials develop non-perovskite slabs in a Sr_4_Fe_6_O_13±δ_ phase keeps them in a broad array of O^2−^ non-stoichiometry^18^. Mixed oxygen-ions and electron conducting oxides have attracted great interest owing to their promising use in ceramic separation filters of hydrocarbons incomplete oxidation and oxygen, fuel cell cathodes, or gas detectors^19–22^.

According to the authors’ best knowledge and after searching international scientific databases, there are a few studies done on a Sr_4_Fe_6_O_13_ system. Little attention has been paid on its magnetic and electrical properties, whereas most studies are focused on its applications in oxygen membranes^23,24^. An auto-combustion method we used in the present work to prepare a stable Sr_4_Fe_6_O_13_ of small crystallites. The as-prepared powder was annealed at different temperatures in order to get a single Sr_4_Fe_6_O_13_ phase. A major objective of this work is to explore magnetic properties in small Sr_4_Fe_6_O_13_ crystallites at and below room temperatures. Further, ac conductivity and dielectric properties are studied on a broad range of frequencies and at different temperatures.

## Results and discussion

### Structural properties

**Figure 1:**
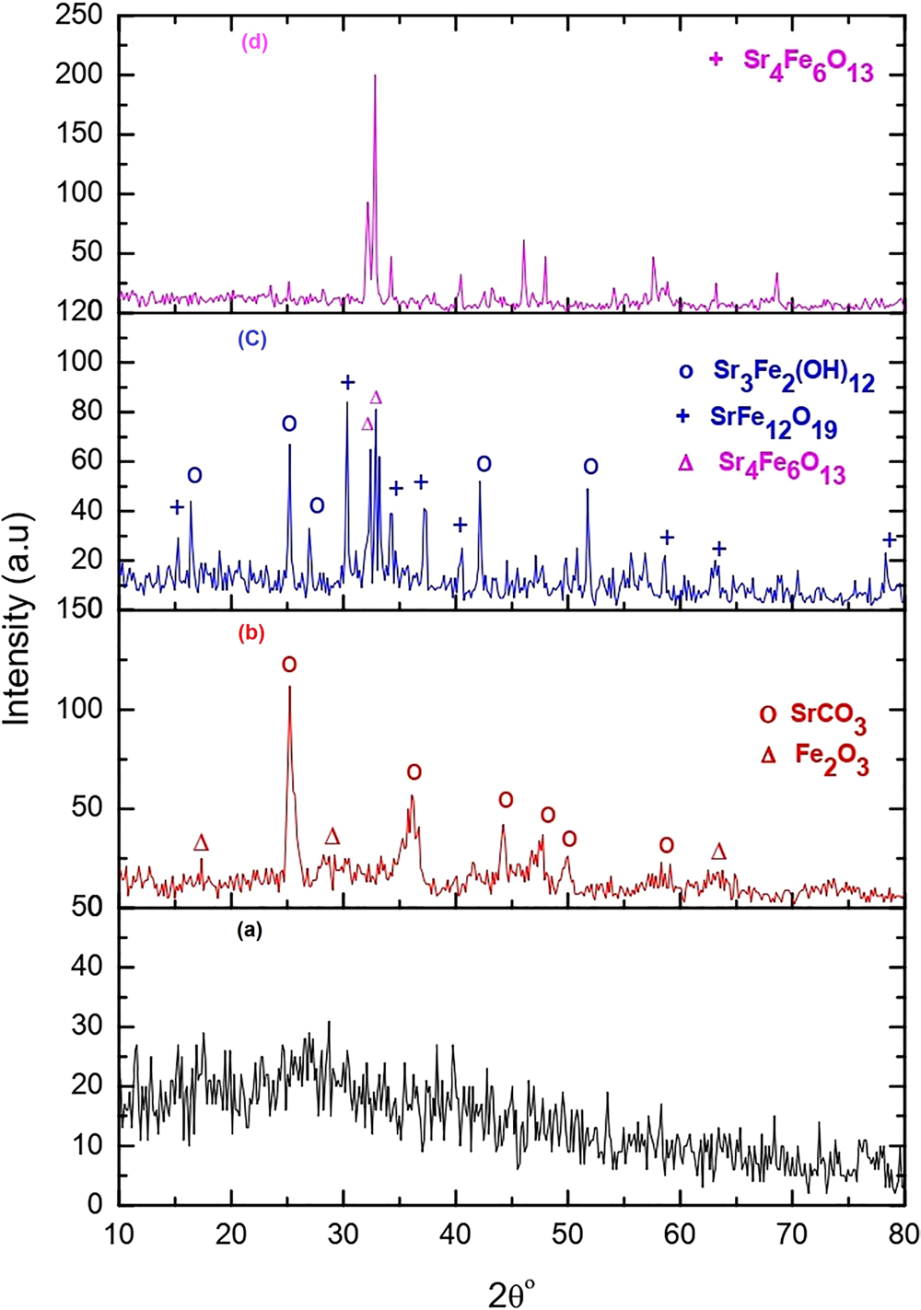
XRD patterns of (**a**) as-prepared (S_0_) and annealed samples at (**b**) 500 °C for 3 h (S_1_), (**c**) 1100 °C for 3 h (S_2_), and (**d**) 1100 °C for 10 h (S_3_).

X-ray diffraction (XRD) patterns of the various samples are shown in Fig. 1. The as-prepared sample S_0_ in Fig. 1a reveals an amorphous pattern with no defined peaks. Figure 1b shows formation of two different phases in the as-prepared sample annealed at 500 °C for 3 h (S_1_). The main phase of an orthorhombic SrCO_3_ accompanies a secondary phase of a rhombohedral α-Fe_2_O_3_ according to JCPDS files 00-005-0418 and 00-073-0603, respectively. The SrCO_3_ forms in a chemical reaction of Sr(NO_3_) with CO_2_ during the synthesis^25^. The diffraction pattern in Fig. 1c of sample S_2_ annealed at 1100 °C for 3 h demonstrates three phases. The main phase orthorhombic Sr_4_Fe_6_O_13_ contains two secondary phases of hexagonal SrFe_12_O_19_ and cubic Sr_3_Fe_2_(OH)_12_. Figure 1d shows XRD of a single phase orthorhombic Sr_4_Fe_6_O_13_ (according to JCPDS file 78-2403) of sample S_3_ annealed at 1100 °C for 10 h. No any secondary phase is observed here. An average crystallite size of the fabricated powder was determined using the Scherrer relation^26,27^.

1 $${D}={k}\lambda {/}{\beta }\,\cos \,{\theta }$$

Here, k is the shape factor (commonly taken approximately as 0.89), λ is wavelength of the X-ray beam used, β is the full-width at half maximum of (251) peak and θ is the diffraction angle. An average D-value of Sr_4_Fe_6_O_13_ was found to be ~72 nm. A microstrain (α) present in the Sr_4_Fe_6_O_13_ crystallites was computed as per a model relation^26,27^,

2 $$\alpha ={\beta }\,\cos \,{\theta }/4$$

A typical α = 0.04 value is found for sample S_3_. A dislocation density δ = 1.92 × 10^17^ lines/cm^2^ (looked as a dislocation line length) contained therein is estimated as^28^,

3 $$\delta =\frac{1}{{{D}}^{2}}$$

**Figure 2:**
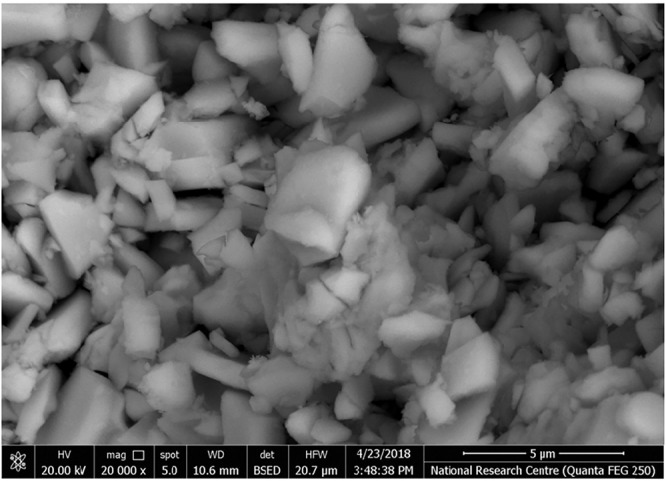
SEM images of sample S_3_.

**Figure 3:**
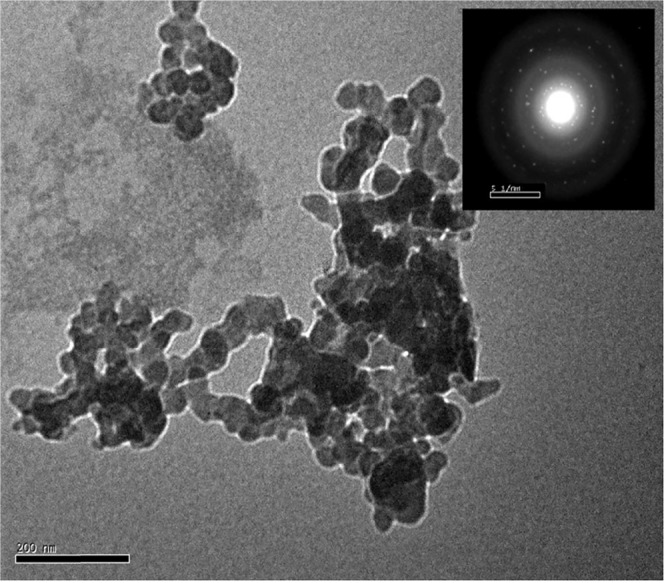
TEM images of sample S_3_, with SAED pattern in the inset.

A scanning electron microscope (SEM) was used to examine morphology of the Sr_4_Fe_6_O_13_ samples as illustrated in Fig. 2. Small grains are observed of irregular rectangular or cubic shapes, with an average size of 85 nm (S_3_) in a good agreement with the D-value estimated from Scherrer relation. The Sr_4_Fe_6_O_13_ images were studied more closely in a transmission electron microscope (TEM) as given in Fig. 3. Small particles are observed with an average 68 nm size, which exhibit electron diffraction rings with spots, characterizing a nanocrystalline phase in agreement with the XRD analysis.

### Magnetic properties

**Figure 4:**
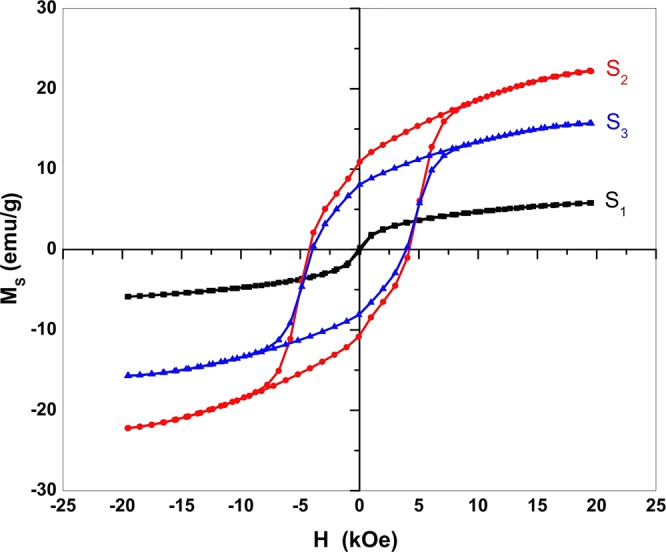
M-H Hysteresis loops for S_1_, S_2,_ and S_3_ samples.

Figure 4 depicts magnetic hysteresis loops of samples S_1_, S_2_, and S_3_ of a ferromagnetic behavior. Values of saturation magnetization (M_s_), remanence magnetization (M_r_), coercivity (H_c_) and squareness ratio (R = M_r_/M_s_) determined from the loops are given in Table 1. Sample S_1_ shows soft ferromagnetic behavior with M_s_ = 5.8 emu/g and H_c_ = 102.0 Oe. The main phase in this sample is SrCO_3_, which has no magnetic moment. A secondary phase Fe_2_O_3_ contributes the magnetic features. Sample S_2_ displays improved M_s_ = 22.2 emu/g, H_c_ = 4223.6 Oe, and R = 0.487 values as it contains a main phase Sr_4_Fe_6_O_13_ with secondary phases Sr_3_Fe_2_(OH)_12_ and SrFe_12_O_19_. The hexagonal ferrite SrFe_12_O_19_ is considered to be made up of alternating spinel (S = Fe_6_O_8_^2**+**^**)** and hexagonal (R = SrFe_6_O_11_^2−^) layers. The O^2−^ ions are closely packed with Sr^2+^ ions in a hexagonal layer and the Fe^3+^ ions distribute in five distinct sites: three octahedral sites (12k, 2a and 4f2), one tetrahedral (4f1) site and one bipyramidal site (2b)^29^. The magnetic structure given by the Gorter model is ferromagnetic with five different sublattices, three parallel (12k, 2a, and 2b) and two anti-parallel (4f1 and 4f2), which are coupled with superexchange interactions through O^2−^ ions^30^. The Sr^2+^ ions are responsible for a large uniaxial magnetic anisotropy controls perturbation of the crystal lattice^31^. Zhang *et al*. demonstrated the Sr_3_Fe_2_(OH)_12_ exhibits a weak ferromagnetic behavior with M_s_ = 0.86 emu/g and H_c_ = 258.43 Oe^32^. So, the magnetic behavior in sample S_2_ is mainly due to the SrFe_12_O_19_ and Sr_4_Fe_6_O_13_ ferrites. Further, sample S_3_ has M_s_ = 15.7 emu/ g, H_c_ = 3956.7 G and R = 0.512 as measured at room temperature.

**Table 1:** Magnetic properties of Sr_4_Fe_6_O_13_ samples prepared in different conditions.

Sample	M_s_ (emu/g)	M_r_ (emu/g)	H_c_ (Oe)	R
S_1_	5.8	0.2	102	0.03^4^
S_2_	22.2	10.8	4223	0.48
S_3_	15.7	8.05^1^	3956	0.51

**Figure 5:**
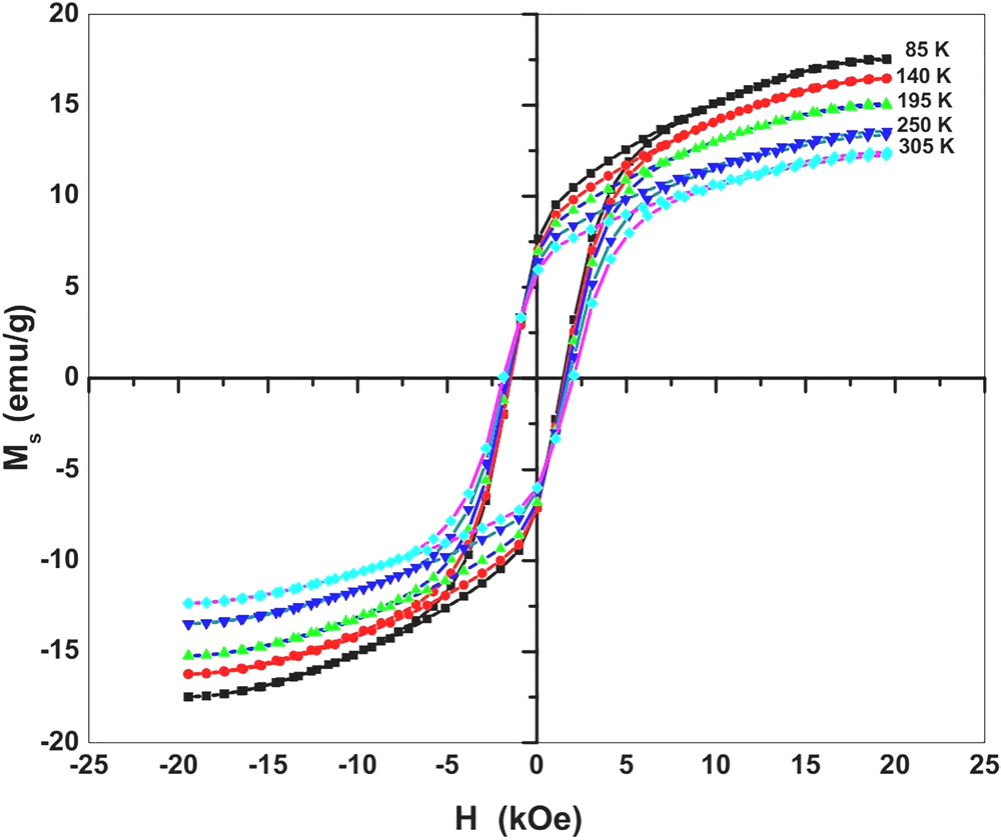
M-H hysteresis loops measured for sample S_3_ at selected temperatures in a range of 85–305 K.

**Figure 6:**
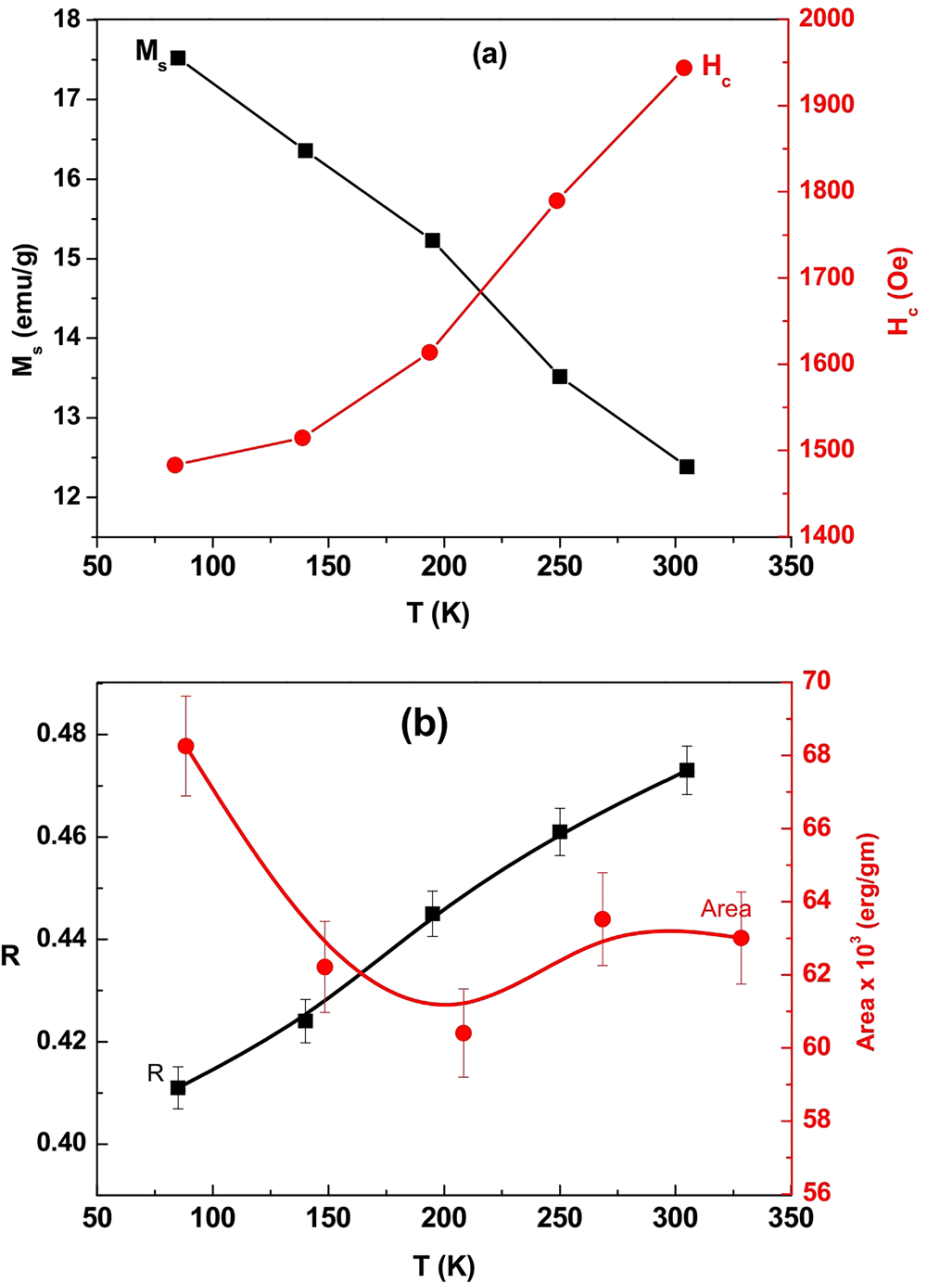
Variations of (**a**) M_s_ and H_c_ and (**b**) squareness (R) and hysteresis area measured against temperature for sample S_3_.

Figure 5 shows hysteresis loops of sample S_3_ measured at different temperatures 85 to 305 K. As shown in Fig. 6a, the M_s_ decreases with increasing temperature, assuming M_s_ = 17.5 emu/g at 85 K relative to M_s_ = 12.4 emu/g at 305 K. This is a typical behavior of ferromagnetic materials in the moment decreases on increasing thermal energy^33,34^. This can be realized by fitting the data using the Bloch’s law^35–37^.

4 $${{M}}_{{s}}({T})={{M}}_{{s}}(0)(1-{B}{{T}}^{{\alpha }})$$

where B is the Bloch constant and M_s_(0) is the M_s_ at 0 K. A value α = 3/2 is assigned according to the mean-field theory for long-range ferromagnetism, with B = 10^−4^–10^−5^ for nanoferrites and 10^−6^ for bulk ferromagnets. The Bloch’s law is valid in our samples^36,38^. Cojocaru studied temperature M_s_ dependence for ferromagnetic nanoparticles in the experimental data follow the Bloch’s law^39^. Figure 6a also shows how H_c_ growing with temperature from 1483 Oe at 85 K to 1944 Oe at 305 K. It is a complicated function of reversal mechanism of spins, anisotropy energy, and magnetic microstructure, viz., shape and size of crystallites, grain boundaries, surfaces, etc. There are also examples in it increasing with temperature^40–42^. Figure 6b shows how R peaks-up with temperature, while the area enclosed in a hysteresis loop decreases up to 195 K and then increases. The area enclosed in a hysteresis cycle represents an irreversible work required to go through the cycle. It depends on dissipation of energy in spin reversals over the fields.

### Dielectric and electrical properties

**Figure 7:**
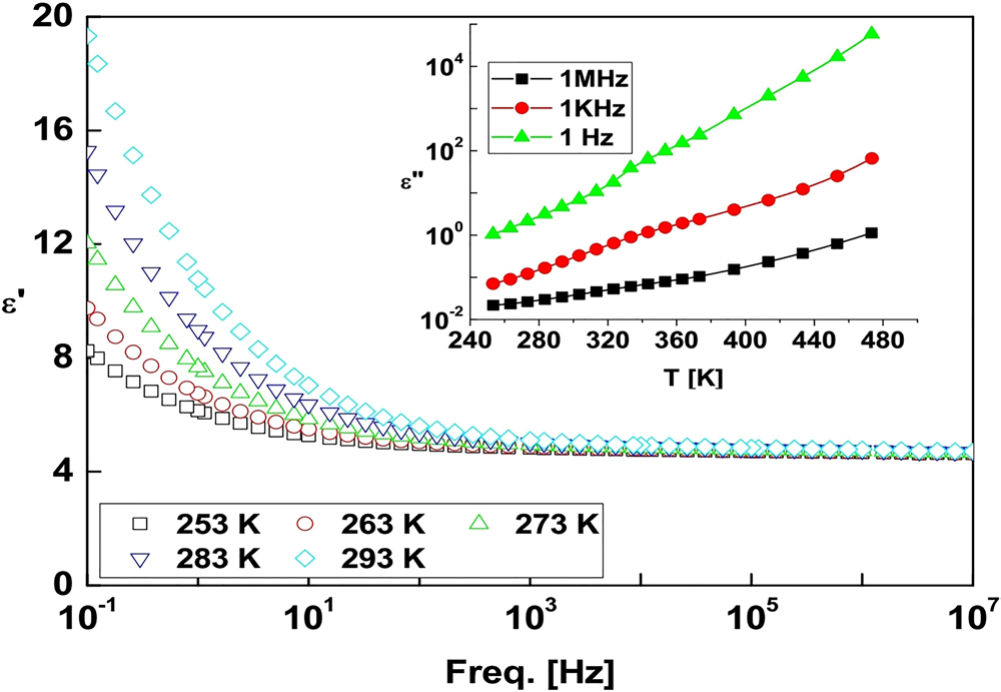
Frequency dependence of ε′-values measured for sample S_0_ at selected temperatures in a range 253–293 K, with temperature ε′′dependence given in the inset at 1 Hz, 1 kHz, and 1 MHz frequencies.

Dielectric permittivity *ε*′ of sample S_0_ varies in two different trends as plotted against frequency in Fig. 7 at different temperatures of 253 to 293 K. In the first region at frequencies above 1 kHz, no remarkable effect of frequency or temperature is noticed. In the second region below 1 kHz up to 0.1 Hz (limited according to the frequency window studied), the ε′ value rises progressively at lower frequencies. As usual^43,44^, the temperature progressively promotes the final ε′ values. Two factors govern enhanced ε′ values at low frequencies; (i) space charge polarization and (ii) interfacial polarization due to domain-wall motion, usually found in multicomponent materials^45–47^. They have induced oscillations compatible to low frequencies of applied external electric fields that facilitate the ε′ values. A field induced charge transport favors conductivity and in turn density and mobility of active charge carriers. It also promotes the interfacial polarization observed here. As oscillations of the charge carriers are reasonably damped, the ε′ value is reduced at higher frequencies. In order to gain a more insight of the effect of temperature on the charge transport even at high frequencies, one has to study separately the effect of temperature on imaginary counter part ε″ of permittivity. The inset in Fig. 7 describes temperature ε″ dependence studied at 10^0^, 10^3^ and 10^6^ Hz frequencies. A resultant value ε*(*ω,T*) = *ε*′(*ω,T*) − *iε*″(*ω,T*) is related to the complex conductivity σ*(*ω,T*) = *σ*′(*ω,T*) + *iσ*″(*ω,T*), with σ*(*ω,T*) = *iωε*_*o*_ε*(*ω,T*), implying *σ*′ *= ε*_*o*_*ωε*″ and *σ*″ *= ε*_*o*_*ωε*′(*ε*_*o*_ = vacuum permittivity).

**Figure 8:**
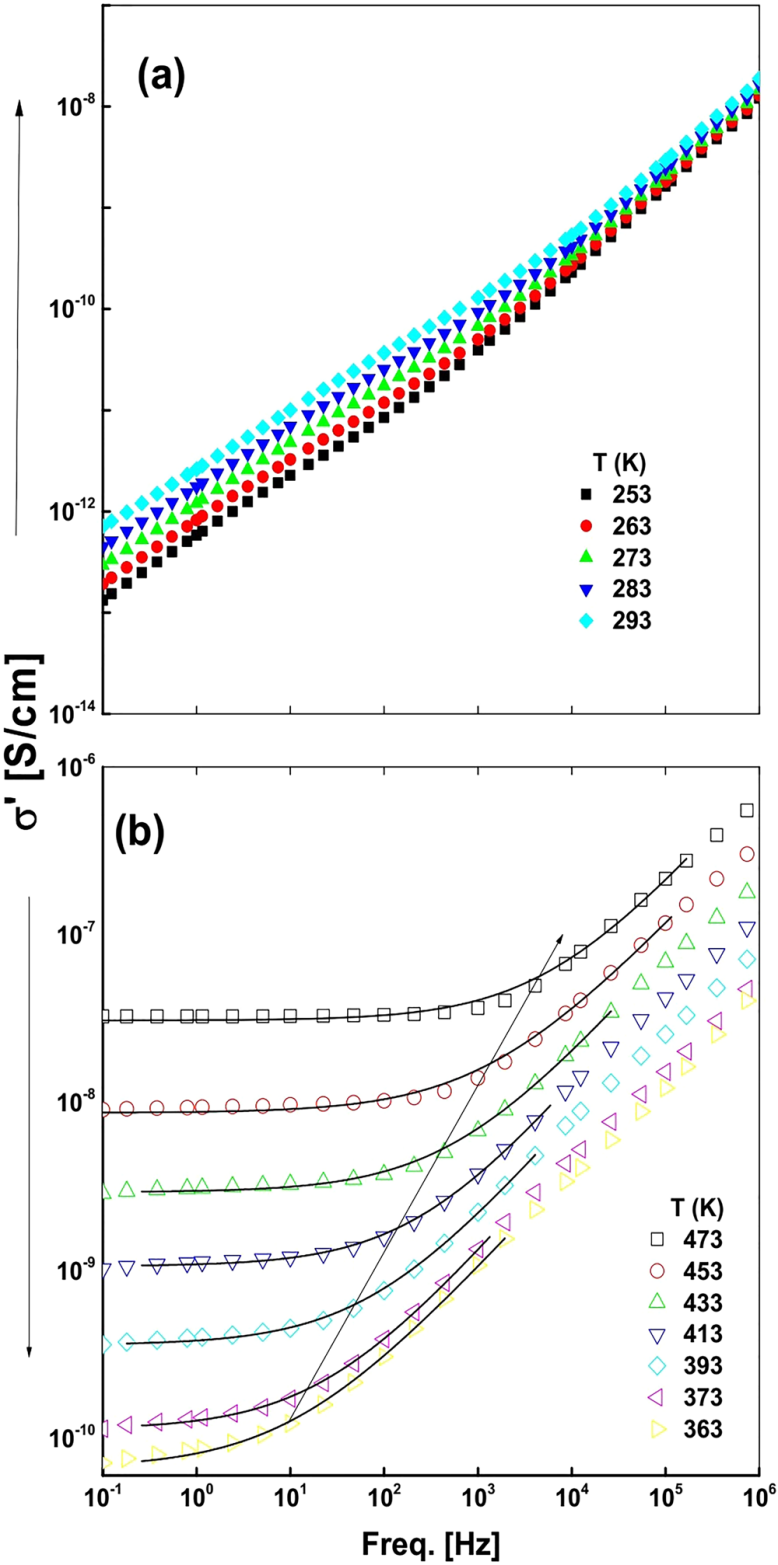
Frequency dependence of σ′-values measured for sample S_0_ at selective temperatures in (**a**) 253–293 K and (**b**) 363–473 K regions.

A gradually increased ε″ value with increasing temperature describes a thermally induced mobility of charge carriers at these frequencies. This confirms a glassy structure of sample S_0_. A linear dependence of conductivity on ε″ explains the results as observed here. A rate of σ′ increase with frequency is slowed down at high frequencies (1 MHz) in a lack of slow dynamic processes. Figure 8 illustrates how σ′ varies over frequencies for a representative sample S_0_ in two temperature regimes. In the 253 to 293 K regime, σ′ gradually decreases in Fig. 8a on lower frequencies, describing a highly insulating material in the ambient temperature in a freezing like behavior of cold charge carriers at low temperatures^47^. It is worth mentioning that, on warming the sample, the conductivity spreads out at low frequencies as the permittivity described in Fig. 7. The frequency σ′ dependence at higher temperatures (Fig. 8b) follows the well-known Jonscher power-law, as prevails in many conductive glasses and polymeric systems^43–45^,

5 $$\sigma {\prime} ={\sigma }_{{dc}}{[1+{\rm{\upsilon }}/{{\rm{\upsilon }}}_{c}]}^{s},{\rm{where}}:(0 < s\le 1)$$

At lower frequencies, a less dependent, or even an independent trajectory (plateau) of frequency, builds up. The plateau yields the dc conductivity σ_dc_ and characteristic frequency ν_c_ in the dispersion of σ′ sets in and turns into a power law at higher frequencies. The σ_dc_ is varied by more than two orders in the 373 to 473 K regime.

**Figure 9:**
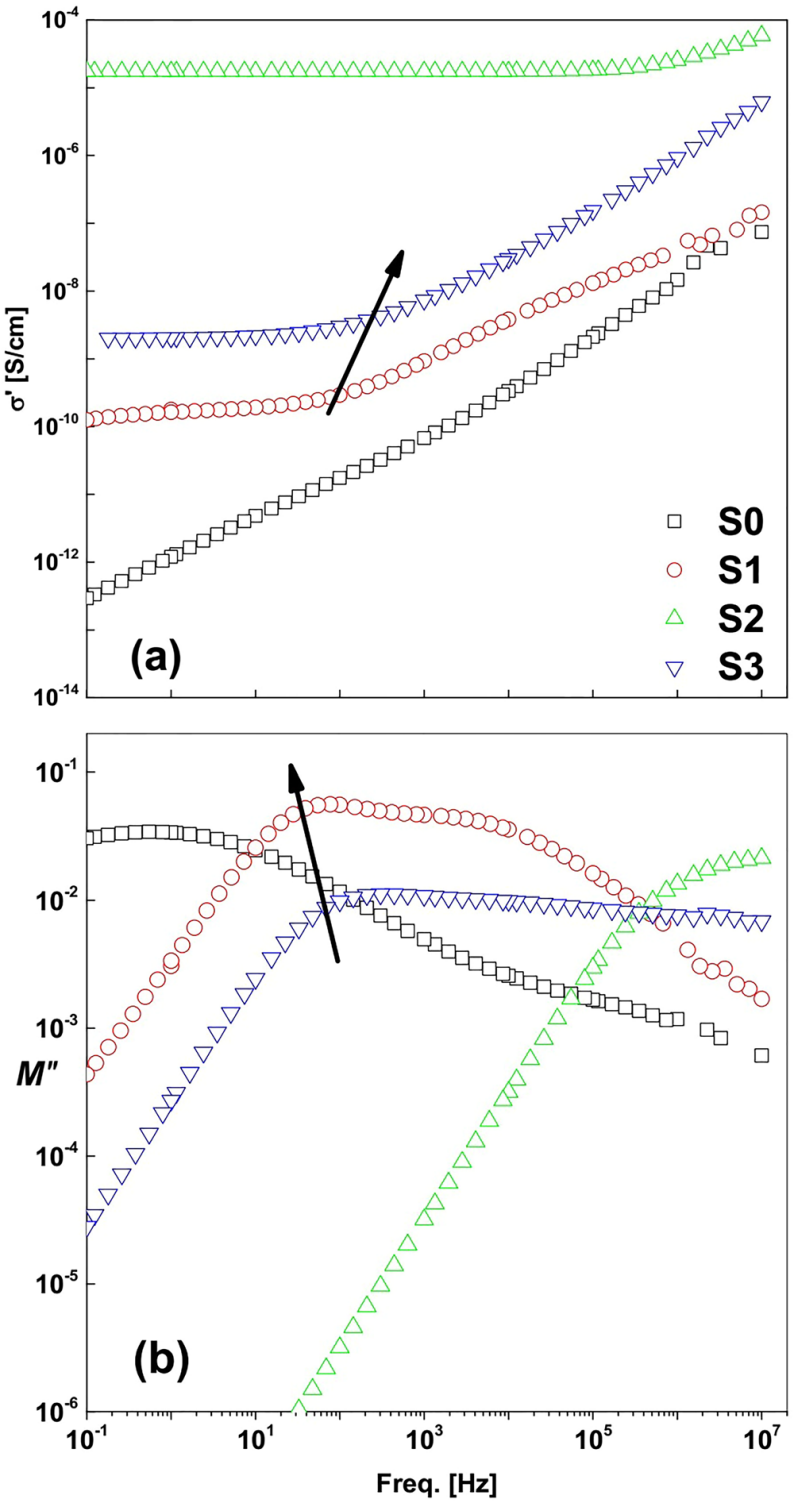
Frequency variations of (**a**) σ′ and (**b**) M′′ values plotted at 273 K for four samples S_0_, S_1_, S_2_ and S_3_ prepared in this investigation.

**Figure 10:**
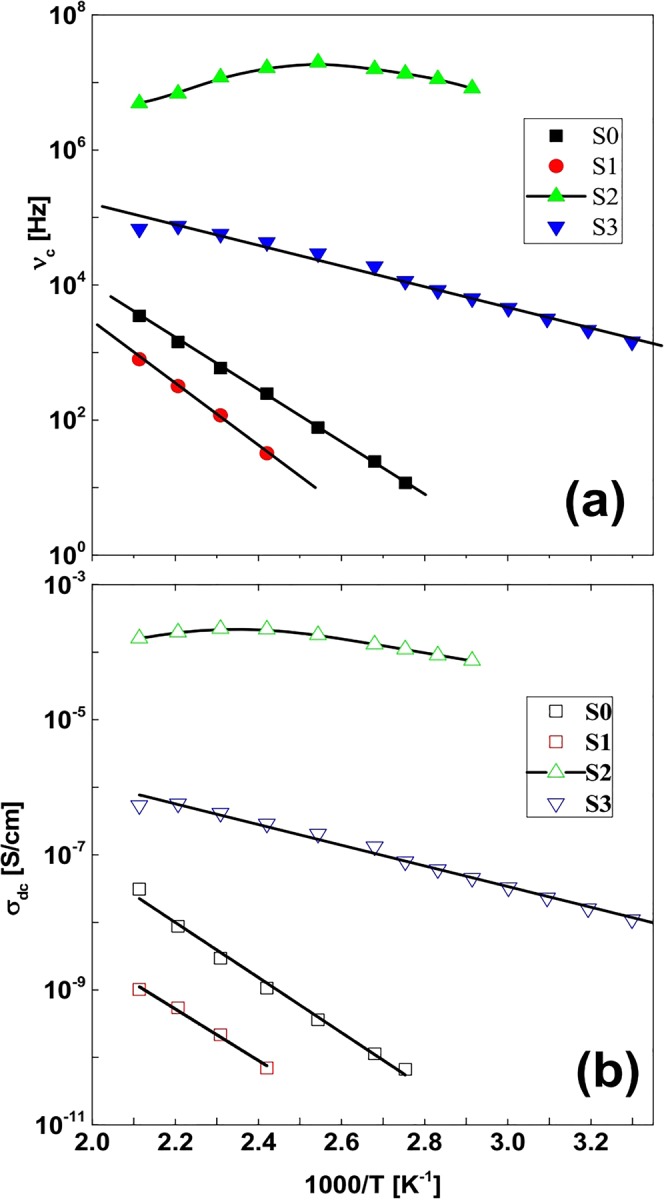
Temperature dependence of (**a**) ν_c_ and (**b**) σ_dc_ values determined by fitting the data in Eq. 5.

Figure 9 depicts σ′ and electric loss modulus ${\rm{M}}{\prime\prime} \left(=\frac{\varepsilon {\prime\prime} }{({\varepsilon {\prime} }^{2}+{\varepsilon }^{{\prime\prime} 2})}\right)$ as a function of frequency at near 0 K for the four samples. Sample S_2_, which contains SrFe_2_O_19_ with a secondary phase Sr_3_Fe_2_(OH)_12_, reveals the highest σ′ value in Fig. 9a at a characteristic frequency (usually called hoping frequency), which is characterized by a maximum peak position in the M″(ν) plot in Fig. 9b. Samples S_0_ and S_1_ peak up σ′ at higher frequencies and it reflects in a small peak in the M″ plots as a result of charges accumulate at potential wells in a kind of interfacial polarization. The charge carriers move short distances accompanied by the relaxation polarization dynamics consistently with what is it is reported earlier^48–50^. Here, a σ_dc_ value is related to the characteristic frequency in the maximum peak position in the M″(ν) plot and not to the peak intensity as marked by the rows. An increasing σ_dc_ with increasing characteristic frequency agrees well with the Barton-Nakajima-Namikawa (BNN) relation correlated between dc and ac conductivities, according to σ_dc_ ≈ ν_c_^51–54^_._ Further, the M″(ν,T) spectra suggest a remarkably enhanced conductivity on generating mono and multiphase structures over an amorphous sample S_0_. Thus, sample S_2_ of three phases has the highest conductivity in the shortest hopping time. Two main parameters of charge transport σ_dc_ and ν_c_ can be deduced by fitting the data in the Jonscher’s universal power law in Eq. 5, with 1 > s > 0.5. Figure 10 plots so obtained values over T^−1^ for the four samples. Almost all data found to follow an Arrhenius relation:

6 $$\log ({S})=\,\log ({{S}}_{\infty })-\frac{\mathrm{ln}\,10{{E}}_{{A}}}{{{K}}_{{B}}{T}}$$

where S could be the dc-conductivity or any other characteristic parameter, *E*_*A*_ is the activation energy and *K*_*B*_ is Boltzmann constant. A value *E*_*A*_ = 78.1 kJ/mol found for sample S_0_ (amorphous) is reduced to be 73.2 kJ/mol for sample S_1_ (of two phases), or 53.0 kJ/mol for sample S_3_ of a single phase. In sample S_2_, which contains three phases, the said plots deviate from the Arrhenius relation, extending a wide peak like behavior. This reflects higher ability of ions to transport in promoted conductivity over other samples. Identical behavior of both parameters in these samples confirms validity of the BNN-relation.

## Experimental work

### Synthesis of samples

Sr_4_Fe_6_O_13_ was synthesized using a citrate auto-combustion method. Ferric nitrate and strontium nitrate were mixed in citric acid in the stoichiometric ratio to get a clear solution. Ratio of metal nitrates to citric acid was 1:1. Ammonia solution was added dropwise until the pH became 7. The mixture was stirred at 600 rpm and slowly evaporated at 130 °C to form a gel. Viscosity and color were changed as sol turned into a brown puffy porous dry gel, which then was ignited to burn in a strong auto-combustion process with evolving gases (CO_2_, NO_2_, NO, NH_3_, CH_4,_ etc.). The as-synthesized powder S_0_ was calcined at 500 °C for 3 h (S_1_) and at 1100 °C for 3 h (S_2_), or 10 h (S_3_), with a heating rate 4 °C/min^55^.

### Measurements and analyses

The structure was examined by an X-ray diffractometer (XRD) of Burker-D8 with Cu-kα radiation of wavelength λ = 1.5418 Å. Morphology and surface shape of the fine particles was analyzed using a field emission scanning electron microscope (model QUANTA-FEG250, Netherlands) and a transmission electron microscope (TEM) of JEOL-1010. The magnetic measurements were performed using a vibrating sample magnetometer (VSM, LakeShore 7410, USA) with applied fields up to 20 kOe at room temperature. The electrical and dielectric properties were studied of the samples on a long-range of frequency (0.1 Hz to 10 MHz) using a powerful broadband dielectric spectrometer, BDS. It is utilizing a high-resolution Alpha analyzer with an active sample head (Novocontrol GmbH concept 40). All measurements were done isothermally at selected temperatures over 223 to 473 K, which were controlled by a Quatro Novocontrol cryo-system with stability better than ±0.1 K. The samples were sandwiched between two gold-plated brass electrodes of 10 or 20 mm in diameter in parallel plate geometry. The control and data acquisition processes were performed by a WINDETA software^56,57^.

## Conclusions

A fine Sr_4_Fe_6_O_13_ powder was synthesized through a sol-gel auto-combustion method and then annealed at 500 °C for 3 h and at 1100 °C for 3 h, or 10 h in finely tuning its yield of small crystallites. A major phase Sr_4_Fe_6_O_13_ forms in 1100 °C annealing, with a secondary phase of SrFe_12_O_19_ and Sr_3_Fe_2_(OH)_12_. A single phase Sr_4_Fe_6_O_13_ appears on prolong anneal to 10 h. It shows hard magnetic behavior with M_s_ = 12.4 emu/g, H_c_ = 3956.7 Oe, and R = 0.512. It slowly loses M_s_ but gains H_c_ on warming over 85 to 305 K in a typical hard magnet. The ac-conductivity follows the well-known Jonscher relation at higher temperatures and it linearly decreases on decreasing frequency at lower temperatures indicating an insulating feature due to freezing of mobility of the charge carriers. The dc-conductivity is related to a characteristic frequency in the Barton-Nakajima-Namikawa relation.

## References

[1] Hoefler SF, Trimmel G, Rath T (2017). Progress on lead-free metal halide perovskites for photovoltaic applications: a review. Monatshefte fur Chemie.

[2] King G, Woodward PM (2010). Cation ordering in perovskites. J. Mater. Chem..

[3] Jing P (2015). Width-controlled M-type hexagonal strontium ferrite (SrFe_12_O_19_) nanoribbons with high saturation magnetization and superior coercivity synthesized by electrospinning. Sci. Rep.

[4] Kanamaru F, Shimada M, Koizumi M (1972). Crystallographic properties of and mössbauer effect in Sr_4_Fe_6_O_13_. J. Phys. Chem. Solids.

[5] Fjellvåg H, Hauback BC, Bredesen R (1997). Crystal structure of the mixed conductor Sr_4_Fe_4_Co_2_O_13_. J. Mater. Chem..

[6] Bredesen R, Norby T, Bardal A, Lynum V (2000). Phase relations, chemical diffusion and electrical conductivity in pure and doped Sr_4_Fe_6_O_13_ mixed conductor materials. Solid State Ionics.

[7] Fossdal A (2001). Phase equilibria and microstructure in Sr_4_Fe_6−x_CoxO_13_ 0≤x≤4 mixed conductors. Solid State Ionics.

[8] Ohkawa M (1997). The structural study of Sr_4_Fe_6_O_13_ using a Weissenberg technique and synchrotron radiation – unusual ionic radii of five-coordinated Fe3+ ions. Zeitschrift für Krist. - Cryst. Mater.

[9] MacChesney JB, Jetzt JJ, Potter JF, Williams HJ, Sherwood RC (1966). Electrical and Magnetic Properties of the System SrFeO_3_-BiFeO_3_. J. Am. Ceram. Soc.

[10] Tang Y (2016). Structural chemistry and magnetic properties of the perovskite Sr_3_Fe_2_TeO_9_. J. Solid State Chem..

[11] Gore SK (2017). The structural and magnetic properties of dual phase cobalt ferrite. Sci. Rep.

[12] Jalli J (2011). Ferrimagnetic Sr_1.5_Ba_0.5_Zn_2_Fe_12_O_22_ (Zn-Y) Single Crystal With Planar Anisotropy. IEEE Magn. Lett.

[13] Patrakeev MV, Mitberg EB, Leonidov IA, Kozhevnikov VL (2001). Electrical characterization of the intergrowth ferrite Sr_4_Fe_6_O_13+δ_. Solid State Ionics.

[14] Pardo JA (2006). Thickness-dependent transport properties of Sr_4_Fe_6_O_13_ epitaxial thin films. Solid State Ionics.

[15] Ma B (1998). Structure and Property Relationships in Mixed-Conducting Sr_4_(Fe_1−x_Cox)_6_O_13±δ_ Materials. J. Solid State Chem..

[16] Yoshiasa, A., Ueno, K., Kanamaru, F. & Horiuchi, H. Structure of Sr_4_Fe_6_O_13_, a new perovskite-derivative in the Sr_4_Fe_6_O_13_ system. *Mater. Res. Bull.* **21**, 175–181 (1986).

[17] Guggilla S, Manthiram A (1997). Crystal Chemical Characterization of the Mixed Conductor Sr(Fe, Co)_[sub 1.5]_O_[sub y]_ Exhibiting Unusually High Oxygen Permeability. J. Electrochem. Soc..

[18] Waerenborgh JC, Avdeev M, Patrakeev MV, Kharton VV, Frade JR (2003). Redox behaviour of Sr_4_Fe_6_O_13±δ_ by Mössbauer spectroscopy and neutron diffraction. Mater. Lett..

[19] Orlovskaya, N. & Browning, N. D. *Mixed ionic electronic conducting perovskites for advanced energy systems*. (Kluwer Academic Publishers, 2004).

[20] Bouwmeester, H. J. M. Chapter 10 Dense ceramic membranes for oxygen separation. In *Membrane Science and Technology* **4**, 435–528 (Elsevier, 1996).

[21] Badwal SPS, Ciacchi FT (2001). Ceramic membrane technologies for oxygen separation. Adv. Mater..

[22] Hashim SS, Mohamed AR, Bhatia S (2011). Oxygen separation from air using ceramic-based membrane technology for sustainable fuel production and power generation. Renew. Sustain. Energy Rev..

[23] Kilner JA (2000). Fast oxygen transport in acceptor doped oxides. Solid State Ionics.

[24] Fisher CAJ, Islam MS (2005). Mixed ionic/electronic conductors Sr_2_Fe_2_O_5_ and Sr_4_Fe_6_O_13_: atomic-scale studies of defects and ion migration. J. Mater. Chem..

[25] Jean M, Nachbaur V, Bran J, Le Breton JM (2010). Synthesis and characterization of SrFe_12_O_19_ powder obtained by hydrothermal process. J. Alloys Compd..

[26] Patterson AL (1939). The scherrer formula for X-ray particle size determination. Phys. Rev.

[27] Zsigmondy R, Scherrer P (1912). Bestimmung der inneren Struktur und der Größe von Kolloidteilchen mittels Röntgenstrahlen. in Kolloidchemie Ein Lehrbuch.

[28] Shintani T, Murata Y (2011). Evaluation of the dislocation density and dislocation character in cold rolled Type 304 steel determined by profile analysis of X-ray diffraction. Acta Mater..

[29] Ram S (1989). Crystallisation of BaFe_12_O_19_ hexagonal ferrite with an aid of B_2_O_3_ and the effects on microstructure and magnetic properties useful for permanent magnets and magnetic recording devices. J. Magn. Magn. Mater..

[30] Smit, J. & Wijn, H. P. J. *Ferrites: physical properties of ferrimagnetic oxides inrelation to their technical applications*. (Wiley, 1959).

[31] Pullar RC (2012). Hexagonal ferrites: A review of the synthesis, properties and applications of hexaferrite ceramics. Progress in Materials Science.

[32] Zhang H, Wang T, Chen X, Zhu W (2016). Controllable hydrothermal synthesis of star-shaped Sr_3_Fe_2_(OH)_12_ assemblies and their thermal decomposition and magnetic properties. Particuology.

[33] Lin CR, Chu YM, Wang SC (2006). Magnetic properties of magnetite nanoparticles prepared by mechanochemical reaction. Mater. Lett..

[34] Chen DH, Chen YY (2001). Synthesis of barium ferrite ultrafine particles by coprecipitation in the presence of polyacrylic acid. J. Colloid Interface Sci..

[35] Franco A, Pessoni HVS, Machado FLA (2015). Spin-wave stiffness parameter in ferrimagnetic systems: Nanoparticulate powders of (Mg, Zn) Fe_2_O_4_ mixed ferrites. J. Appl. Phys..

[36] Vázquez-Vázquez C, López-Quintela MA, Buján-Núñez MC, Rivas J (2011). Finite size and surface effects on the magnetic properties of cobalt ferrite nanoparticles. J. Nanoparticle Res.

[37] Mandal K, Mitra S, Kumar PA (2006). Deviation from Bloch T3/2 law in ferrite nanoparticles. Europhys. Lett..

[38] Nguyet DTT, Duong NP, Hung LT, Hien TD, Satoh T (2011). Crystallization and magnetic behavior of nanosized nickel ferrite prepared by citrate precursor method. J. Alloys Compd..

[39] Cojocaru S (2011). Temperature dependence of magnetization of a nanosize Heisenberg ferromagnet. Optoelectron. Adv. Mater. Rapid Commun.

[40] Nagai N, Sugita N, Maekawa M (1993). Formation of hexagonal, platelike Ba-ferrite particles with low temperature dependence of coercivity. J. Magn. Magn. Mater..

[41] Chagas EF (2014). Thermal effect on magnetic parameters of high-coercivity cobalt ferrite. J. Appl. Phys..

[42] Nihon Butsuri Gakkai. & Ōyō Butsuri Gakkai (2013). Temperature Dependence of Coercivity Behavior in Fe Films on Fractal Rough Ceramics Surfaces. Japanese J. Appl. Phys. JJAP..

[43] Sharma SK, Rajeswari PV, Tiwari B, Ram S (2017). Hydrothermal synthesis of LiMnPO4-C(sp2) hybrids, conductive channels, and enhanced dielectric permittivity: a modulated ionic conductor. Ionics (Kiel)..

[44] Tiwari B, Ram S, Banerji P (2018). Biogenic Synthesis of Tunable Core-Shell C-CaIn_2_O_4_, Interface Bonding, Conductive Network Channels, and Tailored Dielectric Properties. ACS Sustain. Chem. Eng..

[45] Elliott SR (1978). Temperature dependence of a.c. conductivity of chalcogenide glasses. Philos. Mag. B.

[46] Azab AA (2018). Structural and dielectric properties of prepared PbS and PbTe nanomaterials. J. Semicond..

[47] Abd El-Aziz ME, Youssef AM, Kamel S, Turky G (2019). Conducting hydrogel based on chitosan, polypyrrole and magnetite nanoparticles: a broadband dielectric spectroscopy study. Polym. Bull..

[48] Dhankhar S (2016). Electrical conductivity and modulus formulation in zinc modified bismuth boro-tellurite glasses. Indian J. Phys..

[49] Moussa MA (2019). Dielectric investigations and charge transport in PS-PAni composites with ionic and nonionic surfactants. J. Phys. Chem. Solids.

[50] Biswas D (2018). Conductivity spectra of silver-phosphate glass nanocomposites: Frequency and temperature dependency. J. Non. Cryst. Solids.

[51] Turky G, Sangoro JR, Rehim MA, Kremer F (2010). Secondary relaxations and electrical conductivity in hyperbranched polyester amides. J. Polym. Sci. Part B Polym. Phys.

[52] Macdonald, J. R. Universality, the Barton Nakajima Namikawa relation, and scaling for dispersive ionic materials. *Phys. Rev. B - Condens. Matter Mater. Phys*. **71**, (2005).

[53] Sangoro JR (2009). Charge Transport and Dipolar Relaxations in Hyperbranched Polyamide Amines. Macromolecules.

[54] Turky G, Shaaban SS, Schöenhals A (2009). Broadband dielectric spectroscopy on the molecular dynamics in different generations of hyperbranched polyester. J. Appl. Polym. Sci..

[55] Azab, A. A., El-Dek, S. I. & Solyman, S. Unsual features of ferromagnetic/antiferromagnetic nanocomposites. *J. Alloys Compd*. **656** (2016).

[56] Broadband Dielectric Spectroscopy, 10.1007/978-3-642-56120-7 (Springer Berlin Heidelberg, 2003).

[57] Moussa MA (2017). Relaxation dynamic and electrical mobility for poly(methyl methacrylate)-polyaniline composites. J. Appl. Polym. Sci..

